# The prognostic value of the preoperative c-reactive protein/albumin ratio in ovarian cancer

**DOI:** 10.1186/s12885-017-3220-x

**Published:** 2017-04-21

**Authors:** Yubo Liu, Shengfu Chen, Chengyu Zheng, Miao Ding, Lan Zhang, Liangan Wang, Meiqing Xie, Jianhua Zhou

**Affiliations:** 1 Department of Ultrasound, Sun Yat-Sen University Cancer Center, State Key Laboratory of Oncology in South China, Collaborative Innovation Center for Cancer Medicine, 651 Dong feng Road East, Guangzhou, 510060 People’s Republic of China; 2 Department of Obstetrics and Gynecology, Sun Yat-Sen Memorial Hospital of Sun Yat-Sen University, Guangdong Provincial Key Laboratory of Malignant Tumor Epigenetic and Gene Regulation , 107 Yan Jiang Road West, Guangzhou, 510120 People’s Republic of China; 3 Guangdong Provincial Key Laboratory of Orthopedics and Traumatology, The First Affiliated Hospital of Sun Yat-Sen University, Guangzhou, 510700 People’s Republic of China

**Keywords:** C-reactive protein/albumin ratio, Inflammation-based prognostic score, Ovarian cancer, Prognosis

## Abstract

**Background:**

Inflammation plays an important role in the pathogenesis of ovarian cancer. This study sought to investigate the association between the preoperative c-reactive protein/albumin ratio (CRP/Alb) and oncological outcomes in ovarian cancer patients.

**Methods:**

Two hundred patients with histologically verified ovarian cancer between June 2006 and July 2012 were retrospectively reviewed. Overall survival was evaluated by the Kaplan–Meier method and log-rank test. The significance of risk factors for overall survival was evaluated with the Cox proportional hazards model. Additionally, area under the receiver operating characteristic curve (AUC) was used to compare the predictive ability of CRP/Alb, Glasgow Prognostic Score (GPS), modified GPS (mGPS), neutrophil lymphocyte ratio (NLR), platelet lymphocyte ratio (PLR), prognostic index (PI) and prognostic nutritional index (PNI).

**Results:**

The optimal cutoff value of CRP/Alb was 0.68. Increased CRP/Alb (≥0.68) was associated with advanced stage, residual tumor, ascites, elevated serum carbohydrate antigen(CA)-125 level, GPS, and mGPS (all *p* < 0.05). Patients with high CRP/Alb had poor overall survival compared to those with low CRP/Alb (*p* < 0.001). Multivariable analysis showed that CRP/Alb (Hazard Ratio (HR) 1.330, 95% confidence interval (CI) 1.131–1.564, *p* = 0.001), tumor stage (HR 1.577, 95% CI 1.189–2.091, *p* = 0.002), residual tumor (HR 2.337, 95% CI 1.518–3.597, *p* < 0.001) and age (HR 1.017, 95% CI 1.000–1.035, *p* = 0.046) were independent prognostic factors for overall survival. Additionally, the CRP/Alb showed greater AUC values at 1 year (0.692), 3 years (0.659), and 5 years (0.682) than GPS, mGPS and PNI.

**Conclusions:**

The CRP/Alb is a novel independent marker of poor prognosis among ovarian cancer patients and shows superior prognostic ability compared to the established inflammation-based prognostic indices.

**Electronic supplementary material:**

The online version of this article (doi:10.1186/s12885-017-3220-x) contains supplementary material, which is available to authorized users.

## Background

Ovarian cancer has the highest death rate among all gynecological malignancies worldwide [1]. Primary cytoreductive surgery alone or in combination with adjuvant chemotherapy is now widely advocated as the standard treatment for ovarian cancer patients [2]. Nevertheless, despite the improvement in surgical procedures and the development of adjuvant therapy such as platinum-based chemotherapy, neoadjuvant chemotherapy, intraperitoneal hyperthermic therapy and molecular targeted therapies, the long-term survival is still poor [3, 4]. Ovarian cancer is a heterogeneous disease, and the prognosis is variable. Some patients may experience better clinical outcomes than others [5]. Therefore, the identification of factors that could help to predict the prognosis and individualize the treatment according to the stratification of risks may improve the survival of ovarian cancer patients.

In fact, ovarian cancer has been found to be closely related to inflammation [6, 7]. Firstly, ovulation itself is a natural inflammatory process involving ovarian cortex cyclical rupture and healing, which is regarded as an underlying factor of ovarian cancer [8, 9]. Secondly, patients who suffer endometriosis or pelvic inflammatory disease have an increase in the subsequent risk of ovarian cancer [10, 11]. In contrast, oral contraceptives inhibiting ovulation reduce the risk of ovarian cancer [11]. Additionally, tubal ligation or hysterectomy has been proven to offer protection against ovarian cancer by preventing the retrograde spread of proinflammatory factors from the lower genital tract to the ovaries [10–12]. Furthermore, anti-inflammatory therapy can reduce the risk of ovarian cancer and extend the survival of ovarian cancer patients [13, 14]. Given the close relationship between inflammation and ovarian cancer, several inflammation-based prognostic indices have been constructed to predict the clinical outcome. To date, the Glasgow Prognostic Score (GPS) [15], neutrophil lymphocyte ratio (NLR) [16] and platelet lymphocyte ratio (PLR) [17] were reported to display prognostic value in ovarian cancer patients.

The C-reactive protein/albumin ratio (CRP/Alb), consisting of CRP and albumin, was initially used to assess the outcome of patients with acute medical admissions and sepsis [18, 19]. Recently, the prognostic ability of CRP/Alb has been reported in patients with hepatocellular carcinoma [20], gastric cancer [21] and esophageal squamous cell carcinoma [22, 23]. Elevated preoperative CRP/Alb has been associated with the poor survival of patients with the aforementioned cancers. However, up to now, no study has been conducted to clarify the clinical significance and prognostic value of this marker in ovarian cancer.

Therefore, in this study, we retrospectively investigated the impact of preoperative CRP/Alb on the overall survival (OS) in ovarian cancer and compared the predictive value of CRP/Alb, GPS, mGPS, NLR, PLR, prognostic index (PI) and prognostic nutritional index (PNI).

## Methods

### Ethics statement

Written informed consents for their information to be stored and used in the hospital database were obtained prior to data collection, and the study was approved by the ethics committee of the Sun Yat-sen University Cancer Center. The study was conducted in accordance with the Declaration of Helsinki to protect personal data.

### Study population

This retrospective analysis was conducted on patients pathologically diagnosed with ovarian cancer at Sun Yat-sen University Cancer Center in Guangzhou, China, between June 2006 and July 2012. All the patients were included in this study based on the following criteria: (a) histologically confirmed ovarian cancer; (b) available serum CRP and albumin levels at diagnosis; (c) adequate clinico-pathological and follow-up data; (d) no clinical evidence of infection or other inflammatory conditions; and (e) no second malignancies or multiple primary malignancies. Finally, 200 patients diagnosed with ovarian cancer were enrolled in our study. The patients were treated with hysterectomy, bilateral salpingo-oophorectomy, pelvic and/or paraaortic lymphadenectomy, appendectomy, and omentectomy. Patients with stage Ic to IV disease received platinum-based chemotherapy following surgery. Patient charts were reviewed to obtain age, preoperative laboratory measurements, postoperative tumor characteristics and time of death or time of last follow-up from the hospital database at the Sun Yat-sen University Cancer Center and pathological records from the Institute of Pathology at the same institution. OS time was defined as the interval between the date of operation and the date of death or the last follow-up. Patient follow-up was maintained until death or the cutoff date of December 2014. The clinico-pathological and full blood count data before initial treatment were obtained.

### Statistical analysis

Pearson’s χ^2^ test was used to examine the correlations of CRP/Alb value with clinico- pathological parameters. The ROC curve was calculated, and the Youden index (maximum (sensitivity + specificity-1)) [24] was used to determine the optimal cutoff value for CRP/Alb, PNI, NLR, PLR and CA-125. All patients were divided into two different groups (high or low CRP/Alb ratio group) according to the optimal cutoff value of CRP/Alb. The Kaplan–Meier method was used to plot the survival curves, and the log-rank test was used to compare the differences between the subgroups. A univariate and multivariate analysis was performed for the prognostic factors using the Cox proportional hazard model, with significant variables (*p* < 0.05) in univariate mode being further analyzed in the multivariate Cox proportional hazards mode. Area under receiver operating characteristics curve (AUC) analyses were performed using MedCalc statistical software version 15.2.1 (MedCalc Software bvba, Ostend, Belgium). Other analyses were performed using SPSS version 13.0 (Chicago, Illinois, USA). Statistical significance was set at *p* < 0.05 (two-tailed).

## Results

### Demographics

A total of 200 subjects were studied, with a median age of 53 years (range 18–83 years). A total of 110 (55%) patients had an elevated CRP concentration (10 mg/L), and 22 (11%) patients had hypoalbuminemia (albumin <35 g/L) prior to surgery. Of the 22 patients with hypoalbuminemia, 21 (95.45%) had an elevated CRP concentration. According to clinical criteria, almost all the patients (192) received platinum-based chemotherapy.

### ROC analysis

Using the OS rate as an endpoint, when the CRP/Alb, PNI, NLR and PLR were 0.68, 48.80, 2.57 and 165.24, respectively, the Youden index was maximal. Therefore, the optimal cutoff value of the CRP/Alb, PNI, NLR and PLR were set at 0.68, 48, 2.5 and 165, respectively. However, no reasonable cutoff value of CA-125 could be defined to predict survival outcome due to its low specificity (see Additional file 1). The CRP/Alb, GPS, mGPS, NLR, PLR, PI and PNI were constructed as described in Table 1.

**Table 1:** Inflammation-based prognostic scores

Scoring systems	Score
C-reactive protein/albumin (CRP/Alb)	
C-reactive protein/albumin ≤ 0.68	0
C-reactive protein/albumin > 0.68	1
Glasgow Prognostic Score (GPS)	
CRP(≤ 10 mg/L) and albumin(≥ 35 g/L)	0
CRP(≤ 10 mg/L) and albumin(< 35 g/L)	1
CRP(>10 mg/L) and albumin(≥ 35 g/L)	1
CRP(>10 mg/L) and albumin(< 35 g/L)	2
The modified GPS	
CRP(≤ 10 mg/L) and albumin(≥ 35 g/L)	0
CRP(≤ 10 mg/L) and albumin(< 35 g/L)	0
CRP(>10 mg/L)	1
CRP(>10 mg/L) and albumin(< 35 g/L)	2
Neutrophil lymphocyte ratio(NLR)	
Neutrophil count: lymphocyte count < 2.5	0
Neutrophil count: lymphocyte count ≥ 2.5	1
Platelet lymphocyte ratio(PLR)	
plt count: lymphocyte count ≤ 165	0
plt count: lymphocyte count > 165	1
Prognostic nutritional index(PNI)	
Albumin(g/L) + 5 × total lymphocyte count × 10^9^/L ≥ 48	0
Albumin(g/L) + 5 × total lymphocyte count × 10^9^/L < 48	1
Prognostic index(PI)	
CRP(≤ 10 mg/L) and white cell count(≤ 11 × 10^9^/L)	0
CRP(≤ 10 mg/L) and white cell count(> 11 × 10^9^/L)	1
CRP(>10 mg/L) and white cell count(≤ 11 × 10^9^/L)	1
CRP(>10 mg/L) and white cell count(> 11 × 10^9^/L)	2

### Relationship between CRP/alb and clinico-pathological factors

The CRP/Alb ranged from 0.005 to 7.503 with a median of 0.334. A total of 69 patients (34.5%) were categorized as high CRP/Alb (≥0.68), and 131 patients (65.5%) were categorized as low CRP/Alb (<0.68) according to the optimal cutoff value. The elevated CRP/Alb was significantly associated with a more advanced tumor stage (*p* = 0.001), fewer patients with ideal cytoreductive surgery (*p* = 0.049), the presence of ascites (*p* = 0.009) and higher serum CA-125 level (*p* = 0.002). In addition, CRP/Alb was associated with other inflammatory biomarkers, including GPS, mGPS and PLR (all *p* < 0.001), but not with PNI, NLR, and PI (all *p* > 0.05). The relationships between the CRP/Alb and clinico-pathological characteristics are summarized in Table 2.

**Table 2:** The correlation between clinicopathological factors and CRP/Alb ratio in ovarian cancer patients (*n* = 200)

Variable	No. of patients		*P* value
	CRP/Alb <0.68	CRP/Alb ≥0.68	
Age			
≤50 years	59	25	0.23
>50 years	72	44	
Tumor stage			0.001
FIGO I	23	2	
FIGO II	27	6	
FIGO III	63	44	
FIGO IV	18	17	
Grade			
G1	40	15	0.325
G2	52	34	
G3	39	20	
Residual tumor			0.049
≤ 2 cm	94	40	
> 2 cm	37	29	
Histological type			0.552
Serous	76	45	
Mucinous	18	9	
Endometrioid	10	4	
Clear cell	8	6	
Others	19	5	
Ascites			0.009
N0	96	38	
Yes	35	31	
Albumin			<0.001
≤35 g/L	6	18	
>35 g/L	125	51	
CRP level			<0.001
≤ 10 mg/L	90	0	
> 10 mg/L	41	69	
CA-125(U/mL)			0.002
≤ 35	16	0	
> 35	115	69	
GPS(0/1/2)	91/35/5	0/53/16	<0.001
mGPS(0/1/2)	90/37/4	0/53/16	<0.001
PNI(0/1)	100/31	46/23	0.143
NLR(0/1)	110/21	50/19	0.053
PI(0/1/2)	59/58/14	26/37/6	0.452
PLR(0/1)	56/75	9/60	<0.001
Survival(months)	43.12(2.08–104.27)	24.32(0.85–68.79)	<0.001

*FIGO* International Federation of Gynecologists and Obstetricians, *G* grade, *CRP* C-reactive protein, *CA* cancer antigen, *GPS* Glasgow Prognostic Score, *mGPS* modified, *GPS* NLR Neutrophil lymphocyte ratio, *PLR* Platelet lymphocyte ratio, *PI* Prognostic index, *PNI* Prognostic Nutritional Index

### Survival analysis

**Fig. 1:**
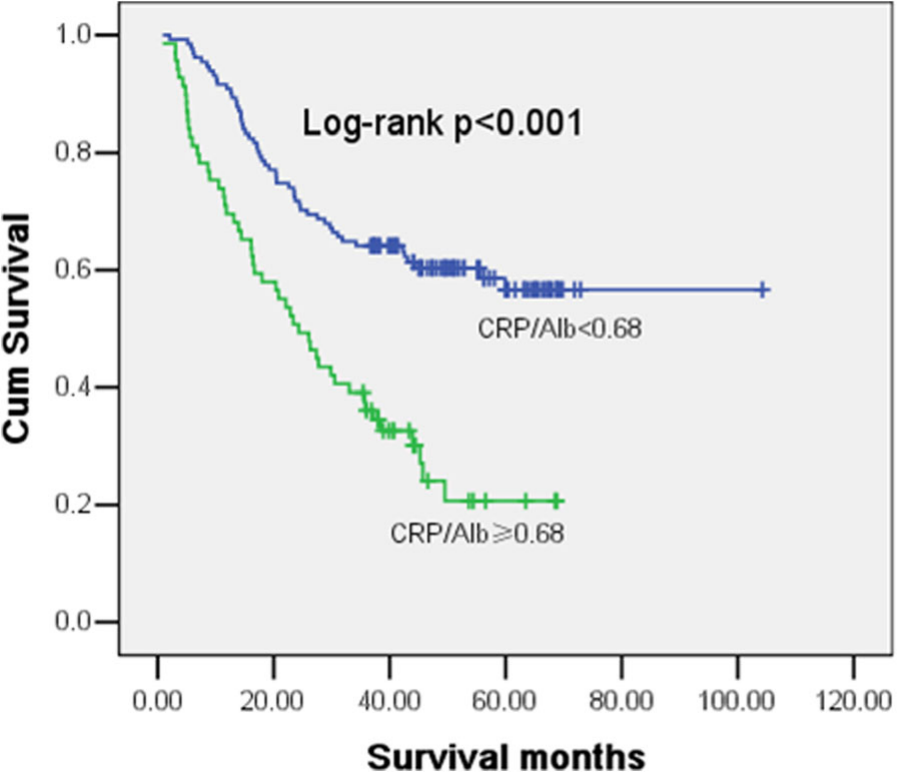
Kaplan–Meier curves showing the difference in OS for patients with ovarian cancer categorized according to the optimal cutoff of CRP/Alb

At the time of analysis, 103 (51.5%) patients had died, and the overall median survival was 37.47 months (range 0.85–104.27 months). Patients with a CRP/Alb < 0.68 had a median survival of 43.12 (range 2.08–104.27) months compared with 24.32 (range 0.85–68.79) months in patients with a CRP/Alb ≥ 0.68 (HR1.287, 95% CI 1.139–1.454, *p* < 0.001). The 1-year, 3-year, and 5-year OS rates were 83.5%, 53.5%, and 15.5%, respectively. Fig. 1 shows the Kaplan–Meier curve for OS and reveals that a high CRP/Alb is a consistent factor for poor prognosis in ovarian cancer patients (*p* < 0.001, log-rank test).

### Impact of inflammatory scores as predictors of OS

By univariate OS analysis, age (*p* = 0.004), CRP/Alb (*p* < 0.001), tumor stage (*p* < 0.001), postoperative residual tumor mass (*p* < 0.001), histological subtype (*p* = 0.015), ascites (*p* = 0.011), CRP (*p* = 0.027), hypoalbuminemia (*p* < 0.001), GPS (*p* = 0.025), mGPS (*p* = 0.018), PLR (*p* = 0.006), PNI (*p* = 0.003) and PI (*p* = 0.028), but not histological grade, CA-125 level or NLR, were associated with postoperative OS. By multivariate analysis adjusted for the effects of all significant variables associated with survival in univariate mode, age (HR1.017 95% CI 1.000–1.035, *p* = 0.046), tumor stage (HR 1.577, 95% CI 1.189–2.091, *p* = 0.002), residual tumor (HR 2.337, 95% CI 1.518–3.597 *p* < 0.001) and CRP/Alb (HR 1.330, 95% CI 1.131–1.564, *p* = 0.001) remained significant independent predictors of OS (see Table 3 **)**.

**Table 3:** Univariate and multivariate analysis of potential prognostic factors for overall survival

Variables	Univariate analysis			Multivariate analysis		
	HR	95% CI	*P*	HR	95% CI	*P*
Age(years) (≤ 50 vs >50)	1.024	1.007–1.040	0.004	1.017	1.000–1.035	0.046
FIGO Stage(I vs II vs III vs IV)	2.028	1.573–2.614	<0.001	1.577	1.189–2.091	0.002
Grade(G1 vs G2 vs G3)	1.176	0.915–1.512	0.206			
Residual tumor (≤2 cm vs >2 cm)	3.352	2.267–4.955	<0.001	2.337	1.518–3.597	<0.001
Histological subtype	0.821	0.700–0.962	0.015			
Ascites(yes vs no)	1.671	1.127–2.479	0.011			
Albumin	0.928	0.889–0.967	<0.001			
CRP level	1.005	1.001–1.009	0.027			
CA-125	1.000	1.000–1.000	0.145			
GPS	1.383	1.042–1.835	0.025			
mGPS	1.409	1.061–1.873	0.018			
PNI	1.787	1.212–2.634	0.003			
NLR	1.407	0.885–2.238	0.149			
PI	1.377	1.035–1.832	0.028			
PLR	1.921	1.207–3.056	0.006			
CRP/Alb ratio	1.287	1.139–1.454	<0.001	1.330	1.131–1.564	0.001

*HR* hazard ratio, *FIGO* International Federation of Gynecologists and Obstetricians, *G* grade, *CRP* C-reactive protein, *CA* cancer antigen, *GPS* Glasgow Prognostic Score, *mGPS* modified, *GPS* PNI Prognostic Nutritional Index, *NLR* Neutrophil lymphocyte ratio, *PI* Prognostic index, *PLR* Platelet lymphocyte ratio, *Alb* Albumin

### Comparison of the predictive ability

**Fig. 2:**
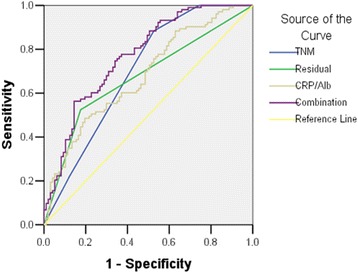
The receiver operating curves analysis of CRP/Alb, tumor stage, residual tumor and the combination of them to prediction of overall survival

AUC values were used to compare the predictive ability between the CRP/Alb ratio and the other inflammation-based prognostic scores, such as GPS, mGPS, PI, PNI, PLR and NLR (See Table 4). The CRP/Alb showed greater AUC at one year (0.692), three years (0.659), and five years (0.682) compared with the GPS (1 year: *p* = 0.0003, 3 years: *p* = 0.0002 and 5 years: *p* = 0.0190), mGPS (1 year: *p* = 0.0004, 3 years: *p* < 0.0001 and 5 years: *p* = 0.0176) and PI (1 year: *p* = 0.0087, 3 years: *p* = 0.0001 and 5 years: *p* = 0.0101). Similar results were also found in NLR, PLR and PNI, but the difference was not significant (*p* > 0.05), except the comparison between CRP/Alb and NLR at 3 years (*p* = 0.0166). Furthermore, we analyzed the prognostic value of CRP/Alb combined with tumor stage and residual tumor (see Fig. 2). The combination of three parameters displayed greater AUC than any one of them, although the difference was not significant (*p* > 0.05) except for the comparison to the residual tumor (*p* = 0.0271).

**Table 4:** Comparison of the diagnostic performance of several inflammation-based prognostic indices in predicting mortality

Indices	One year follow-up		Three years follow-up		Five years follow-up	
	AUC(95% CI)	*P*	AUC(95% CI)	*P*	AUC(95% CI)	*p*
CRP/Alb	0.692(0.623–0.755)	<0.001	0.659(0.589–0.724)	<0.001	0.682(0.613–0.746)	<0.001
GPS	0.594(0.523–0.663)	0.054	0.579(0.508–0.649)	0.030	0.606(0.535–0.674)	0.040
mGPS	0.596(0.525–0.665)	0.049	0.573(0.502–0.643)	0.046	0.605(0.534–0.673)	0.043
NLR	0.613(0.542–0.681)	0.026	0.567(0.496–0.637)	0.078	0.601(0.530–0.670)	0.089
PNI	0.658(0.588–0.723)	0.002	0.637(0.566–0.704)	<0.001	0.631(0.560–0.698)	0.016
PLR	0.609(0.538–0.677)	0.028	0.595(0.524–0.664)	0.018	0.646(0.575–0.712)	0.007
PI	0.612(0.541–0.680)	0.022	0.569(0.497–0.638)	0.062	0.594(0.522–0.662)	0.063

Comparisons between AUCs at one year: CRP/Alb vs. GPS: *p* = 0.0003; CRP/Alb vs. mGPS: *p* = 0.0004. CRP/Alb vs. PI: *p* = 0.0087

Comparisons between AUCs at three year: CRP/Alb vs. GPS: *p* = 0.0002; CRP/Alb vs. mGPS: *p* < 0.0001. CRP/Alb vs. PI: *p* = 0.0001

CRP/Alb vs. NLR: *p* = 0.0166

*AUC* area under the curve, *CRP/Alb* C-reactive protein/albumin, *GPS* Glasgow Prognostic Score, *mGPS* modified GPS, *NLR* neutrophil to lymphocyte ratio, *PLR* Platelet lymphocyte ratio, *PI* Prognostic index, *PNI* Prognostic Nutritional Index

## Discussion

The present study demonstrated that increased CRP/Alb predicted the poor prognosis of OS in ovarian cancer patients. Moreover, compared to the established inflammation-based prognostic indices GPS, mGPS and PNI, CRP/Alb displayed superior prognostic ability. These results are consistent with previous studies identifying CRP/Alb as predictors of outcome in hepatocellular carcinoma [20], gastric cancer [21] and esophageal squamous cell carcinoma [22, 23].

C-reactive protein (CRP) is an important acute phase response protein produced mainly by hepatocytes, whose levels rise in response to inflammation [25]. Mc Sorley et al. reported that high circulating CRP levels may subsequently promote ovarian cancer [26]. This conclusion was supported by a meta-analysis [27]. Hefler et al. found that elevated CRP is associated with chemical resistance and poor survival in patients with ovarian cancer [28]. The underlying mechanism is that CRP can accelerate angiogenesis based on increased circulating levels of vascular growth factors and circulating interleukin in cancer patients [29, 30].

Albumin is also produced by the liver, which helps to maintain intravascular oncotic pressure, facilitate the transport of substances and scavenge free radicals. It is now considered an indicator of malnutrition. Hypoalbuminemia is related to a sustained systemic inflammatory response, either from the tumor itself or as a host response [31]. Several studies have suggested that the progression of hypoalbuminemia is secondary to the serum elevation of CRP, as many cancer patients with hypoalbuminemia already have increased serum CRP levels [32, 33]. Malnutrition, in turn, is related to poor prognosis in patients with cancer [34]. Several previous studies reported that pre-operative low serum albumin levels are an independent predictor of poor survival in ovarian cancer [35, 36]. Improvement in nutritional status is associated with better survival in ovarian cancer [14] and other tumors [37]. One of the reasons is that the presence of a systemic inflammatory response and the concomitant nutritional decline reduces patient tolerance to treatment toxicities and patient compliance with active treatment [38].

CRP/Alb, which is obtained from the combination of CRP and albumin, may reflect both the inflammatory and nutritional state in cancer patients. Therefore, the presence of a chronic systemic inflammatory response and progressive nutritional decline is reflected by the elevated CRP/Alb, ultimately resulting in reduced survival.

Previous studies have established some possible prognostic factors in ovarian cancer. In particular, the adverse effect of postoperative residual tumor mass, tumor grade, peritoneal dissemination, and histological subtype on patients’ OS has been found in previously published studies [39]. By univariate analysis, we have shown that tumor stage, postoperative residual tumor, histological subtype, ascites, CRP, hypoalbuminemia and age, as well as CRP/Alb, GPS, mGPS, PLR, PNI and PI, are predictors of OS in ovarian cancer. However, by using a Cox regression model of multivariate analysis, we found that only CRP/Alb remained as an independent prognostic marker for poor survival in patients with ovarian cancer along with residual disease, tumor stage and age, suggesting that CRP/Alb has a substantial impact on patient outcome. Surprisingly, albumin is no longer an independent predictor of OS, and age is a marginally significant predictor of OS (*p* = 0.046), conflicting with a recent study [36]. The reason for this difference is that our study included CRP/Alb. In fact, when CRP/Alb was excluded, albumin became an independent risk factor for OS (data not shown), indicating that CRP/Alb is a more powerful predictor than preoperative albumin. We noted that CRP/Alb correlated significantly with advanced tumor stage, residual tumor, increased CA-125 levels and the presence of ascites, suggesting that increased CRP/Alb may correlate with a more aggressive disease phenotype.

On established prognostic factors, postoperative residual tumor mass and ovarian tumor stage have been shown to be the most reliable predictors of outcome in ovarian cancer [40]. Similar to other studies, while CRP/Alb was a significant predictor of OS in patients with ovarian cancer, residual tumor mass and tumor stage remained significantly more powerful predictors of survival, as the Hazard Ratio for residual tumor and tumor stage were 2.337 and 1.577, respectively, compared with the HR of 1.330 for CRP/Alb by multivariate analysis. Further, we analyzed the prognostic value of CRP/Alb combined with tumor stage and residual tumor mass. The combined effect was greater than the individual effect of either variable alone, indicating that CRP/Alb may be the complementary factor for tumor stage and residual tumor mass in predicting the survival in patients with ovarian cancer.

The prognostic significance of preoperative CA-125 levels in ovarian cancer remains controversial at present. A few publications have described an association between CA-125 levels before surgery and survival. Paramasivam et al. reported patients with early-stage ovarian cancer and a preoperative serum CA-125 more than 30 U/mL were significantly associated with impaired survival [41]. Similar results were found by Kumar et al. [42]. However, some studies failed to show a correlation between preoperative CA-125 and prognosis. Mury et al. concluded that the specificity of CA-125 to predict surgical outcome is low, and the prognostic value is questionable [43]. Chi et al. reported that preoperative CA-125 did not predict the primary cytoreductive outcome of patients with advanced ovarian cancer [44]. In the present study, we also could not define a reasonable cutoff value of CA-125 to predict survival outcome due to its low specificity. In addition, although CA-125 was correlated to overall survival in univariate analysis, it was no longer an independent predictor in multivariable analysis. Therefore, we inferred that the main value of CA-125 may be useful in monitoring disease recurrence instead of prognosis.

In addition, it is important to examine whether a new prognostic system is at least equivalent or superior to other current validated prognostic scoring systems. We, therefore, compared the prognostic ability of CRP/Alb with other established inflammation-based prognostic scores, such as GPS, mGPS NLR, PI, PLR and PNI. In the context of ovarian cancer, AUC analysis has shown that CRP/Alb was superior to other inflammation-based prognostic scores in terms of predictive accuracy, which is consistent with several previous studies in hepatocellular carcinoma [20], gastric cancer [21] and esophageal squamous cell carcinoma [22, 23].

To the best of our knowledge, this is the first study to investigate whether CRP/Alb is useful for predicting postoperative outcome in ovarian cancer patients, and we analyzed all seven of these parameters for the first time. Our results suggest that preoperative serum CRP/Alb might serve as a potentially clinically valuable marker in patients with ovarian cancer. Firstly, CRP/Alb has the advantage of being simple to measure, routinely available and well standardized. Secondly, increased CRP/Alb might correlate with a more aggressive disease phenotype, possibly using it to screen a subset of patients with bad prognosis requiring intense therapy. Thirdly, CRP/Alb displays superior prognostic ability compared to other inflammation-based scoring systems. Finally, CRP/Alb may be a complementary factor for tumor stage and residual tumor mass in predicting the survival in patients with ovarian cancer.

Although the present study shows the strong independent prognostic value of the CRP/Alb in ovarian cancer patients, the retrospective nature and the relatively small sample size from a single center should be acknowledged as potential limitations. However, the strict inclusion and exclusion criteria and the level of statistical significance achieved for the prognostic traits tested in our series leave little doubt about the reliability and reproducibility of our findings.

## Conclusion

Preoperative CRP/Alb derived from routine blood tests is an independent prognostic marker in patients with ovarian cancer. Moreover, compared to other inflammation-based prognostic scores, it shows superior prognostic ability. Therefore, clinically, CRP/Alb may be used as a complementary factor to stratify ovarian cancer patients into different prognostic groups for tailored treatment.

## Additional file

Additional file 1: ROC analysis of CA-125 to predict an “optimal” cutoff value (AUC area under the curve). (TIFF 23 kb)

## Abbreviation

Alb: Albumin
AUC: Area under receiver operating characteristics curve
CA-125: Carbohydrate antigen-125
CI: Confidence interval
CRP: C-reactive protein
CRP/Alb: C-reactive protein/albumin ratio
FIGO: International federation of gynecologists and obstetricians
G: Grade
HR: Hazard ratio
mGPS: The modified glasgow prognostic score
NLR: Neutrophil lymphocyte ratio
OS: Overall survival
PI: Prognostic index
PLR: Platelet lymphocyte ratio
PNI: Prognostic nutritional index
