# Supplementary figures and images for: The prognostic value of the preoperative c-reactive protein/albumin ratio in ovarian cancer
Source: BMC Cancer. 2017 Apr 21;17:285. doi: 10.1186/s12885-017-3220-x (PMC5399817; doi:10.1186/s12885-017-3220-x)

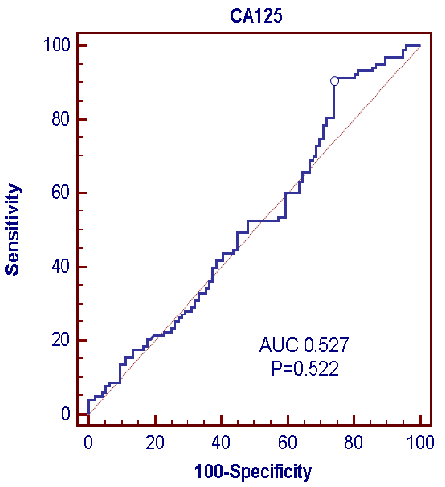

Supplement: Additional file 1: — ROC analysis of CA-125 to predict an “optimal” cutoff value (AUC area under the curve). (TIFF 23 kb) [file 12885_2017_3220_MOESM1_ESM.tif]
